# Changes in Access to Comprehensive Clinical Microbiology Laboratory Testing in Tennessee From 2019–2024

**DOI:** 10.1093/ofid/ofag061

**Published:** 2026-02-11

**Authors:** Kaleb Wolfe, Stephanie A Hart, Stacy Curry-Johnson, Ayesha Khan, Garrett S Booth, Russell B Bowden, William D Dupont, W Dale Plummer, Romney Humphries, Joesph R Wiencek

**Affiliations:** Division of Infectious Diseases, University of Kansas Medical Center, Kansas City, Kansas, USA; Pathology, Microbiology and Immunology, Vanderbilt University Medical Center, Nashville, Tennessee, USA; Geographic Information Systems Lab, Vanderbilt University Libraries, Nashville, Tennessee, USA; Pathology, Microbiology and Immunology, Vanderbilt University Medical Center, Nashville, Tennessee, USA; Pathology, Microbiology and Immunology, Vanderbilt University Medical Center, Nashville, Tennessee, USA; Tennessee Department of Health Laboratory Services, Nashville, Tennessee, USA; Department of Biostatistics, Vanderbilt University Medical Center, Nashville, Tennessee, USA; Department of Biostatistics, Vanderbilt University Medical Center, Nashville, Tennessee, USA; Pathology, Microbiology and Immunology, Vanderbilt University Medical Center, Nashville, Tennessee, USA; Pathology, Microbiology and Immunology, Vanderbilt University Medical Center, Nashville, Tennessee, USA

**Keywords:** laboratory consolidation, geographic access, health equity, social determinants of health, clinical microbiology

## Abstract

**Background:**

Timely clinical microbiology laboratory testing is crucial for effective infectious disease management, impacting antibiotic selection, patient outcomes, and health care costs. This study aimed to evaluate changes in geographic access to comprehensive (Tier 1) clinical microbiology laboratories in Tennessee between 2019 and 2024.

**Methods:**

With the help of the Tennessee Department of Health, Tier 1 microbiology laboratories in Tennessee were identified for both 2019 and 2024. Catchment areas were calculated using a 25-mile drive-time radius around each laboratory. Changes in the number and location of Tier 1 laboratories were quantified, and demographic data were compared between laboratories that stayed open and those that closed.

**Results:**

The number of Tier 1 microbiology laboratories in Tennessee decreased by 38% over the 5-year period, and this resulted in a 35% decrease in square-mile coverage across the state. Covariates associated with laboratory closure included rurality, lower income, higher poverty, race, higher disability, and less education.

**Conclusions:**

A substantial decline in the number of Tier 1 clinical microbiology laboratories in Tennessee between 2019 and 2024 has decreased local access to comprehensive testing in areas with at-risk patient populations. This may have implications for timely diagnosis, appropriate antimicrobial therapy, and, ultimately, patient outcomes. Further research is needed to evaluate the clinical and economic consequences of these changes in laboratory access.

Microbiology testing is critical for the workup of infectious disease patients. For each patient sample, the clinical microbiology laboratory incubates the specimen then performs a culture, gram stain, organism identification, and antimicrobial susceptibility testing (AST) [1]. This testing allows for early detection and identification of causative organisms, clarification of potential sources of infection, and recommendations for appropriate antibiotic therapy [2–5]. Previous studies have shown that empiric antibiotics are often inappropriate and associated with increased mortality, length of stay, and hospital costs [5–8]. Alternatively, appropriate antibiotic therapy guided by microbiology testing is associated with decreased mortality, treatment failure, length of stay, cost, and number of antibiotic agents utilized [2, 3, 7, 9]. In sum, effective care in patients with infectious diseases hinges on access to this testing.

Access to microbiology services, however, is undergoing changes due to various pressures on the health care system, including changing population dynamics, disease prevalence, and economic factors [10]. These pressures have led to the consolidation of clinical laboratories to increase standardization, improve efficiency, reduce costs, and address the shortage of qualified personnel [11, 12]. While consolidation can offer laboratory benefits such as 24/7 staffing, increased standardization, expanded microbiology test menus, and potentially decreased turnaround times, it also presents logistical challenges transporting samples to centralized locations including specimen integrity, time-sensitive tests, chain of custody, temperature control, accurate labeling, and risk management of biohazardous materials [11, 13, 14]. In addition, delays in specimen processing, viability loss of fastidious pathogens during transport, and delayed availability of gram stain results, which in turn inhibits their use in acute care decisions, remain important areas of concern [15]. Some health care facilities have mitigated these challenges through new technology such as rapid molecular assays. At their current stage, however, these tests do not replace blood cultures [16, 17], and their accessibility and utility at the local level remain areas of investigation.

Consolidation also impacts the clinical care team model [18]. As microbiology laboratory personnel and infectious disease (ID) teams are increasingly separated geographically, the traditional relationship between the 2 has suffered. In their recent survey of ID specialists, Pentella et al. found that most providers felt that having an on-site laboratory was critical for timely diagnosis [18]. Often, respondents indicated that their primary laboratory was off-site, with many laboratories located >30 minutes away from the hospital, making it difficult for ID providers to visit the microbiology lab or discuss cases with microbiologists [18].

Unfortunately, few studies have evaluated how these changes affect patient outcomes, and even fewer have looked at how social determinants of health intersect with these barriers to disproportionately affect our most vulnerable populations. In a recent study of an influenza epidemic, for example, Zipfel et al. found that individuals in low-income areas disproportionately bore the burden of disease, with geographic disparities and reduced access to health care further widening these inequities [19]. The authors postulated that these changes were due in part to different contact structures, reduced sickness absenteeism, and systemic barriers to care [19]. There have been even fewer studies on how the changing laboratory landscape has affected patient access to these services. This study investigated recent trends in microbiology laboratory consolidation in Tennessee (TN) to determine their effects on access to laboratory services.

## METHODS

### Ethical Considerations

This study was exempt from institutional review board (IRB) review as it exclusively utilized publicly available data that did not include identifiable private information.

### Study Participants

A list of microbiology laboratories was generated from a preexisting database of sentinel laboratories maintained by the Tennessee Department of Health (TDH) Division of Laboratory Services for the purpose of reporting key communicable diseases [20]. This list is updated annually by the TDH, and laboratories are stratified into tiers by how much of the microbiology identification and susceptibility testing process is performed in-house. The tier systems used by TDH were developed based on the Laboratory Response Network's definition of the Sentinel Laboratory. These laboratories are certified to perform high-complexity testing under the Clinical Laboratory Improvement Amendments of 1988 (CLIA). In-house testing capabilities must include gram staining as well as at least 1 of the following: lower respiratory tract cultures, wound cultures, or blood cultures [21]. For the purposes of this definition, culture testing capability includes incubation, identification, and susceptibility testing. A Tier 1 laboratory meets all components of the above definition (Table 1). If additional testing such as mass spectrometry or next-generation sequencing is outsourced for difficult diagnostic cases, this does not affect the laboratory's tier. This classification system does not include mycology, mycobacteriology, or parasitology. In conjunction with the outreach coordinator at TDH, a member of the research team contacted laboratories to assess if they performed microbiology testing. This 2024 list was then compared with the list from 2019 to determine which laboratories had stopped performing in-house microbiology testing over this time period.

**Table 1.** Definition of Laboratory Tiers per the Laboratory Response Network's Definition of the Sentinel Laboratory

Tier	Laboratory Capabilities
Tier 1	Performs in-house gram staining and any 1 of the following: Blood cultures Lower respiratory tract cultures Wound cultures
Tier 2	Outsources at least 1 of the following processes: Gram staining Blood cultures Lower respiratory tract cultures Wound cultures
Tier 3	Outsources all of the following processes: Gram staining Blood cultures Lower respiratory tract cultures Wound cultures

### Data Collection

To assess factors associated with Tier 1 microbiology laboratory closures within Tennessee, laboratory region, population density, ownership, network affiliation, and profit status were collected. Estimates of the total population for Tennessee in both of the study years were obtained from the US Census Bureau's database [22]. Based on laboratory addresses, corresponding US Census track county data were recorded for median household income, percent poverty, racial demographics, percent uninsured, and wi-fi access [23].

### Geospatial Methods

The initial phase of the geospatial component of the study involved geocoding the locations of clinical laboratories across Tennessee. Addresses were standardized and converted into spatial data using a geocoding service integrated into a Geographic Information System (GIS) platform. This step ensured an accurate representation of each clinic's location, which is critical for subsequent spatial analysis.

To assess the service coverage of each site by year (2019 and 2024), a 25-mile drive-time buffer was generated around each geocoded location. Unlike simple Euclidean (straight line) buffers, drive-time buffers consider road networks, travel speeds, and topographic constraints, offering a more realistic depiction of the accessible area. The analysis utilized network analysis tools within GIS software, which models travel distances based on road infrastructure and typical driving conditions.

To further evaluate the spatial distribution of microbiology laboratories per year, a catchment analysis was conducted, focusing on 2 main metrics: area and population coverage. The total land area within each catchment zone was measured yearly based on available labs. Demographic data, sourced from the US Census Bureau, were intersected with the catchment zones to estimate the population served by the labs. This analysis was performed for different time periods (2019 and 2024), enabling an assessment of temporal changes in population coverage and identification of underserved areas.

The final coverage analysis was visualized using GIS mapping tools. The resulting maps integrated various layers, including clinic locations, drive-time buffers, and population data, to provide a comprehensive view of service accessibility. Areas with limited or no coverage were identified, highlighting potential gaps in service distribution.

### Statistical Methods

Demographics were compared between laboratories that stayed open and those that closed. For normally distributed variables, means, standard deviations, and 2-sided *t* tests were performed using Stata (StataCorp, College Station, TX, USA). Fisher exact tests were used to compare categorical variables (GraphPad Software, Dotmatics, Boston, MA, USA). A 2-sided *P* value <.05 was considered statistically significant. Categorical response variables were analyzed using Hotelling's *t* test [24]. We categorized race as White, Black, Asian, Hispanic/Latino, or other. Education was categorized as less than high school, high school or equivalent, or college.

## RESULTS

### Changes in Tier 1 Microbiology Laboratory Access in Tennessee

In 2019, there were 71 Tier 1 microbiology laboratories across the state, with 23 in Middle, 30 in East, and 18 in West Tennessee. There were a total of 44 Tier 1 microbiology laboratories in 2024, with 17, 15, and 12 in Middle, East, and West Tennessee, respectively. By 2024, 32 of the 71 (45%) 2019 laboratories had closed, and 5 new laboratories had opened, which represents a net loss of 27 laboratories. This corresponds to a 38% decrease in Tier 1 microbiology laboratories across Tennessee over 5 years (Table 2).

**Table 2.** Demographics of Tier 1 Microbiology Laboratories in Tennessee in 2019 and 2024

		2019	2024
Tennessee Total Population		6 862 615	7 227 750
		Tier 1 Microbiology Labs in 2019 (n = 71)	Tier 1 Microbiology Labs in 2024 (n = 44)
Region	East, No. (%)	30 (42)	15 (34)
	West, No. (%)	18 (25)	12 (27)
	Middle, No. (%)	23 (32)	17 (39)
Population density	Rural, No. (%)	25 (35)	10 (23)
	Urban, No. (%)	46 (65)	34 (77)
Network status	Network, No. (%)	61 (86)	34 (77)
	Independent, No. (%)	10 (14)	10 (23)
Ownership	Nonprofit, No. (%)	34 (48)	18 (41)
	Government, No. (%)	14 (20)	10 (23)
	Corporation, No. (%)	23 (32)	16 (36)
Public	No. (%)	14 (20)	12 (27)
Private	No. (%)	57 (80)	32 (73)
Income	Average median household income (SD), %	57 043 (10 961)	59 427 (11 508)
Poverty	Average % poverty (SD)	15 (3)	14 (3)
Race demographics	Average % White (SD)	74 (20)	68 (21)
	Average % Black (SD)	16 (18)	22 (19)
Education	Average % high school educated (SD)	88 (4)	90 (2)
	Average % college (SD)	28 (10)	32 (10)
Insurance status	Average % uninsured (SD)	12 (2)	12 (2)
Disability status	Average % disability (SD)	12 (4)	10 (3)
Wi-fi access	Average % wi-fi access (SD)	83 (5)	84 (5)

**Figure 1.**
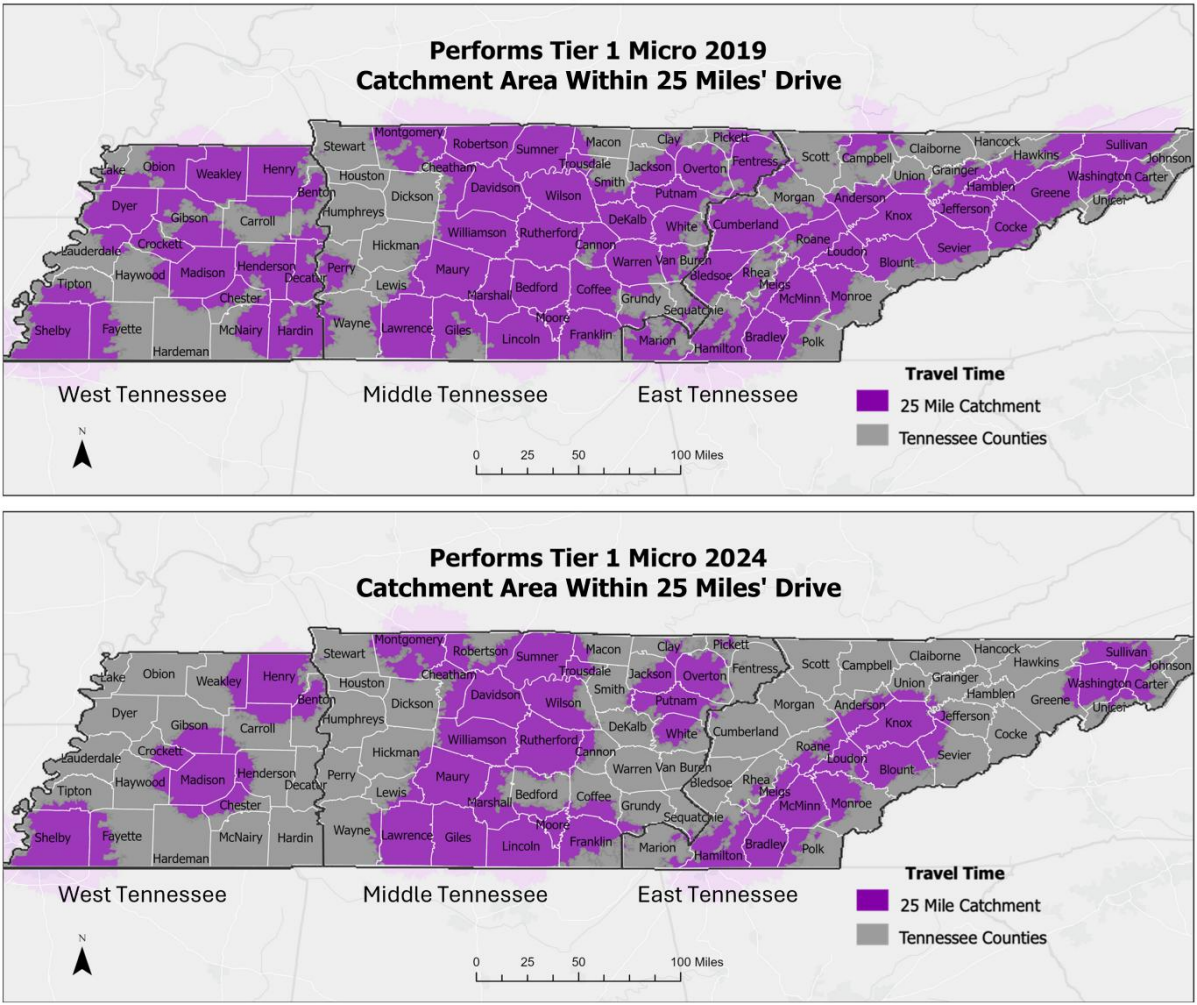
Comparison of catchment areas within 25 miles’ driving distance of a Tier 1 microbiology laboratory. Tier 1 denotes that a laboratory can perform all microbiology services locally. Catchment areas were created using drive-time buffers through a Geographic Information System platform. Top, Catchment areas in 2019. Bottom, Catchment areas in 2024.

When calculating the percentage of the state within a 25-mile catchment area of a Tier 1 microbiology laboratory, 68.5% of the state was within the 25-mile catchment area in 2019. By 2024, only 42% of the state was within the 25-mile catchment area, which corresponds to a 35% decrease in square miles of the state (Figure 1).

### Comparison of 2019 Tier 1 Microbiology Laboratories That Closed vs Remained Open in 2024

When comparing the 2019 Tier 1 microbiology laboratories that closed vs those that remained open in 2024, laboratories in rural areas were significantly associated with closing (*P* = .006). In addition, laboratory closures were significantly associated with lower income (*P* = .019), higher percent poverty (*P* = .026), race (*P* < .001), higher uninsured rate (*P* = .033), higher disability rates (*P* < .001), lower wi-fi access (*P* = .010), and education (*P* < .001). Laboratory closures were also associated with higher percentage of White individuals (*P* = .001) and lower rates of high school (*P* < .001) and college (*P* < .001) education (Table 3).

**Table 3.** Comparison of 2019 Tier 1 Microbiology Laboratories That Closed vs Remained Open by 2024

…		Tier 1 Microbiology Laboratories		*P* Value
		Closed (n = 32)	Remained Open (n = 39)	
Region	East, No. (%)	17 (53)	13 (33)	.147
	West, No. (%)	7 (22)	11 (28)	.594
	Middle, No. (%)	8 (25)	15 (38)	.309
Population density	Rural, No. (%)	17 (53)	8 (21)	**.006**
Network status	Network, No. (%)	30 (94)	31 (79)	.102
Ownership	Nonprofit, No. (%)	18 (56)	16 (41)	.238
	Government, No. (%)	5 (16)	9 (23)	.553
	Corporation, No. (%)	9 (28)	14 (36)	.612
Public vs private	Public, No. (%)	5 (16)	9 (23)	.553
Income	Average median household income (SD), $	53 806 (8674)	59 699 (11 995)	**.019**
Poverty	Average % poverty (SD)	16 (4)	14 (3)	**.026**
Race demographics	Average % White (SD)	82 (16)	67 (21)	**.001**
	Average % Black (SD)	9 (14)	22 (19)	**.003**
Education	Average % high school educated (SD)	87 (4)	90 (2)	**<.001**
	Average % college (SD)	22 (9)	32 (10)	**<.001**
Insurance status	Average % uninsured (SD)	13 (2)	12 (2)	**.033**
Disability status	Average % disability (SD)	13 (4)	10 (2)	**<.001**
Wi-fi access	Average % wi-fi access (SD)	81 (5)	84 (5)	**.010**

Bold values indicate a statistically significant result as determined by a *P* value <0.05.

## DISCUSSION

Concerns over laboratory consolidation have existed for decades, with many arguments being made surrounding its negative impacts [15]. With a goal of reducing operational costs, laboratory consolidation risks achieving this goal at the expense of patient care, particularly for those who are most vulnerable. However, more research is needed to better characterize this phenomenon. Our study highlights a concerning trend in laboratory closures. Between 2019 and 2024, 32 of Tennessee's microbiology laboratories closed, while only 5 new laboratories opened in that period, equating to a 38% decrease over 5 years. Furthermore, the percentage of Tennessee's population residing within a 25-mile catchment area of a Tier 1 microbiology laboratory dropped from 68.5% in 2019 to 42% in 2024, representing a 35% reduction in coverage, all while Tennessee grew nearly 4.6% over a similar time period [23]. Rural locations were particularly impacted, and laboratories that closed were geographically associated with lower income, higher poverty levels, higher uninsured rates, lower wi-fi access, and less education. These findings underscore the urgent need for more research to better understand the full extent of how these trends impact patient outcomes and exacerbate existing inequities.

Our study does have its limitations. To maintain the independence assumption, the above analyses had to be performed at the level of the individual laboratory rather than at the county level. This makes it difficult to assess the effects of laboratory density in counties where multiple laboratories are present on the above results. Additionally, because the study could not distinguish between full laboratory closures and partial outsourcing of microbiology services, we were unable to differentiate these outcomes in our results. It should be noted that the degree of access lost in these communities will likely depend on whether a specific laboratory closed completely or remained open but outsourced its microbiology testing. Finally, while concerns of increased turnaround times with microbiology outsourcing exist within the literature [15], no study to date has been able to evaluate the effect of these closures on measurable health outcomes of patients. Thus, the reduction in Tier 1 laboratory catchment areas demonstrated in this study is a surrogate measure that requires more investigation before it can be definitively said to represent loss of access to microbiology services.

Despite the challenges outlined, the negative effects of laboratory consolidation are not inevitable. Targeted policy interventions have the potential to mitigate these effects and protect patients' access to critical diagnostic services.

***Workforce Development*. **A critical shortage of medical laboratory professionals has significantly contributed to consolidation trends with evidence of increasing vacancies and lack of qualified applicants [12, 25, 26]. Solutions include increasing compensation, investing in training programs, and subsidizing work in underserved areas to incentivize professionals to remain in or relocate to rural communities.

***Increase Reimbursements*. **Chronic underfunding exacerbates the challenges laboratories face. Each year, laboratories are asked to do more while receiving less compensation for their work [15]. Legislative efforts such as the Saving Access to Laboratory Services Act (SALSA), aiming to empower the Centers of Medicare and Medicaid Services to collect private market data from all laboratory segments to ensure fair reimbursements, may represent a potential solution [27].

***Promoting New “Point-of-Need” Technology*. **Point-of-care technologies, such as direct-from-blood molecular testing, currently implemented in some advanced laboratories may allow critical diagnostics to remain local. Subsidization could play a key role in making these advanced technologies more widely available to help ensure equitable access to high-quality diagnostics across all patient populations.

Regardless of the methods used, action must be taken to ensure that all our patients have access to critical diagnostics.

## References

[1] Tabak Y, Vankeepuram L, Ye G, Jeffers K, Gupta V, Murray PR. Blood culture turnaround time in U.S. acute care hospitals and implications for laboratory process optimization. J Clin Microbiol 2018; 56:e00500–18. 30135230 10.1128/JCM.00500-18 PMC6258864

[2] Barenfanger J, Drake C, Kacich G. Clinical and financial benefits of rapid bacterial identification and antimicrobial susceptibility testing. J Clin Microbiol 1999; 37:1415–8. 10203497 10.1128/jcm.37.5.1415-1418.1999 PMC84789

[3] Berild D, Mohseni A, Diep LM, Jensenius M, Ringertz SH. Adjustment of antibiotic treatment according to the results of blood cultures leads to decreased antibiotic use and costs. J Antimicrob Chemother 2006; 57:326–30. 16387751 10.1093/jac/dki463

[4] French K, Evans J, Tanner H, Gossain S, Hussain A. The clinical impact of rapid, direct MALDI-ToF identification of bacteria from positive blood cultures. PLoS One 2016; 11:e0169332. 28036369 10.1371/journal.pone.0169332 PMC5201237

[5] Vallés J, Rello J, Ochagavía A, Garnacho J, Alcalá MA. Community-acquired bloodstream infection in critically ill adult patients: impact of shock and inappropriate antibiotic therapy on survival. Chest 2003; 123:1615–24. 12740282 10.1378/chest.123.5.1615

[6] Diamantis S, Rioux C, Bonnal C, et al Suitability of initial antibiotic therapy for bloodstream infections and the potential role of antibiotic management teams in improving it. Eur J Clin Microbiol Infect Dis 2012; 31:1667–71. 22134774 10.1007/s10096-011-1491-8

[7] Raman G, Avendano E, Berger S, Menon V. Appropriate initial antibiotic therapy in hospitalized patients with gram-negative infections: systematic review and meta-analysis. BMC Infect Dis 2015; 15:395. 26423743 10.1186/s12879-015-1123-5 PMC4589179

[8] Shorr AF, Micek ST, Welch EC, Doherty JA, Reichley RM, Kollef MH. Inappropriate antibiotic therapy in gram-negative sepsis increases hospital length of stay. Crit Care Med 2011; 39:46–51. 20890186 10.1097/CCM.0b013e3181fa41a7

[9] Yokota PKO, Marra AR, Martino MDV, et al Impact of appropriate antimicrobial therapy for patients with severe sepsis and septic shock—a quality improvement study. PLoS One 2014; 9:e104475. 25375775 10.1371/journal.pone.0104475 PMC4222820

[10] Thomson S, Figueras J, Evetovits T, et al Economic Crisis, Health Systems and Health in Europe: Impact and Implications for Policy. World Health Organization, Regional Office for Europe Copenhagen; 2015.

[11] Vandenberg O, Durand G, Hallin M, et al Consolidation of clinical microbiology laboratories and introduction of transformative technologies. Clin Microbiol Rev 2020; 33:e00057–19. 32102900 10.1128/CMR.00057-19 PMC7048017

[12] Leber AL, Peterson E, Dien Bard J. The hidden crisis in the times of COVID-19: critical shortages of medical laboratory professionals in clinical microbiology. J Clin Microbiol 2022; 60:e0024122. 35658527 10.1128/jcm.00241-22 PMC9383190

[13] Lone R, Alhumaidan HSO, Alatoom A, et al Microbiology laboratory consolidation: optimizing diagnostic efficiency and service quality – a case study from Dubai Health, United Arab Emirates. IJID Reg 2025; 17:100779. 41312196 10.1016/j.ijregi.2025.100779 PMC12648958

[14] Simons CC, Capraro GA. The pros and cons of centralizing microbiology services. Available at: https://myadlm.org/cln/articles/2019/julyaug/the-pros-and-cons-of-centralizing-microbiology-services.

[15] Sautter RL, Thomson RB. Point-counterpoint: consolidated clinical microbiology laboratories. J Clin Microbiol 2015; 53:1467–72. 25253793 10.1128/JCM.02569-14 PMC4400739

[16] O’Grady NP, Alexander E, Alhazzani W, et al Society of critical care medicine and the Infectious Diseases Society of America guidelines for evaluating new fever in adult patients in the ICU. Crit Care Med 2023; 51:1570–86.37902340 10.1097/CCM.0000000000006022

[17] Rapszky GA, Do To UN, Kiss VE, et al Rapid molecular assays versus blood culture for bloodstream infections: a systematic review and meta-analysis. eClinicalMedicine 2025; 79:103028. 39968206 10.1016/j.eclinm.2024.103028 PMC11833021

[18] Pentella M, Weinstein MP, Beekmann SE, Polgreen PM, Ellison RT. Impact of changes in clinical microbiology laboratory location and ownership on the practice of infectious diseases. J Clin Microbiol 2020; 58:e01508–19. 32075902 10.1128/JCM.01508-19 PMC7180265

[19] Zipfel CM, Colizza V, Bansal S. Health inequities in influenza transmission and surveillance. PLoS Comput Biol 2021; 17:e1008642. 33705381 10.1371/journal.pcbi.1008642 PMC7951825

[20] TN Department of Health . Reportable diseases: 2023 reporting guidance. Available at: https://www.tn.gov/health/cedep/reportable-diseases.html. Accessed June 13, 2023.

[21] American Society for Microbiology . Laboratory Response Network (LRN) Sentinel Level Clinical Laboratory Protocols. 2013 ed. American Society for Microbiology; 2013.

[22] United States Census Bureau . *County Population Totals and Components of Change: 2019–2024*. US Census Bureau; **2025**.

[23] United States Census Bureau . Quick facts: Tennessee. Available at: https://www.census.gov/quickfacts/fact/table/TN/PST045222.

[24] Wichern DW, Johnson RA, Johnson R. Applied Multivariate Statistical Analysis. 5th ed. Prentice Hall; 2002.

[25] Garcia E, Kundu I, Kelly M, Soles R. The American Society for Clinical Pathology 2022 vacancy survey of medical laboratories in the United States. Am J Clin Pathol 2024; 161:289–304. 37936416 10.1093/ajcp/aqad149

[26] Garcia E, Kundu I, Kelly M, Soles R, Mulder L, Talmon GA. The American Society for Clinical Pathology's job satisfaction, well-being, and burnout survey of laboratory professionals. Am J Clin Pathol 2020; 153:470–86. 32080719 10.1093/ajcp/aqaa008

[27] Saving Access to Laboratory Services Act . HR2377. 118th Congress (2023-2024) ed. **2023**.

